# The other 96%: Can neglected sources of fitness variation offer new insights into adaptation to global change?

**DOI:** 10.1111/eva.12447

**Published:** 2016-12-20

**Authors:** Evatt Chirgwin, Dustin J. Marshall, Carla M. Sgrò, Keyne Monro

**Affiliations:** ^1^ Centre for Geometric BiologyMonash UniversityMelbourneVICAustralia; ^2^ School of Biological SciencesMonash UniversityMelbourneVICAustralia

**Keywords:** evolution, *Galeolaria*, larval development, marine invertebrates, maternal environmental effects, nonadditive genetic effects, temperature

## Abstract

Mounting research considers whether populations may adapt to global change based on additive genetic variance in fitness. Yet selection acts on phenotypes, not additive genetic variance alone, meaning that persistence and evolutionary potential in the near term, at least, may be influenced by other sources of fitness variation, including nonadditive genetic and maternal environmental effects. The fitness consequences of these effects, and their environmental sensitivity, are largely unknown. Here, applying a quantitative genetic breeding design to an ecologically important marine tubeworm, we examined nonadditive genetic and maternal environmental effects on fitness (larval survival) across three thermal environments. We found that these effects are nontrivial and environment dependent, explaining at least 44% of all parentally derived effects on survival at any temperature and 96% of parental effects at the most stressful temperature. Unlike maternal environmental effects, which manifested at the latter temperature only, nonadditive genetic effects were consistently significant and covaried positively across temperatures (i.e., parental combinations that enhanced survival at one temperature also enhanced survival at elevated temperatures). Thus, while nonadditive genetic and maternal environmental effects have long been neglected because their evolutionary consequences are complex, unpredictable, or seen as transient, we argue that they warrant further attention in a rapidly warming world.

## 1 Introduction

Anthropogenic global change is causing populations to encounter changes in selection above natural rates and scales (Davis, Shaw, & Etterson, 2005; Merilä & Hendry, 2014). Populations can evade extinction by one or a combination of three mechanisms: migration to more favorable habitats, phenotypic plasticity, or adaptive evolution (Holt, 1990; Williams, Shoo, Isaac, Hoffmann, & Langham, 2008). The relative importance of each mechanism will vary among species according to their life histories and the timescale considered (Gienapp, Teplitsky, Alho, Mills, & Merila, 2008). For instance, migration is only feasible for species with an alternative habitat and sufficient dispersal capacity to reach it (Hughes, 2000). Furthermore, phenotypic plasticity is predicted to be vital for populations enduring short‐term fluctuations in selection, whereas long‐term directional selection pressures are predicted to require phenotypic responses beyond the limits of plasticity alone (Munday, Warner, Monro, Pandolfi, & Marshall, 2013; Reusch, 2014). Given the limitations of migration and phenotypic plasticity, the ability of many populations to withstand the impacts of global change may largely depend on adaptive evolution.

The adaptive evolution of any natural population requires that individuals vary in fitness, and that this variation has a genetic basis. Phenotypic variation in fitness constrains the evolution of fitness itself and the intensity of selection that acts on any trait (Arnold & Wade, 1984; Crow, 1958). The genetic component of this variation in turn constrains the rate at which fitness increases from generation to generation: The greater the genetic variance in fitness, the faster the evolution of fitness and of traits that are correlated with it (Fisher, 1958). For the most part, studies that have explored the capacity for populations to adapt to future scenarios of global change have focused on the additive genetic variance in fitness (Merilä & Hendry, 2014), which predicts the phenotypic effects of alleles independently of their specific genetic background (Falconer & Mackay, 1996). Thus, additive genetic effects account for the fraction of fitness variation that is known to be inherited stably from one generation to the next, forming the basis of evolutionary responses to selection. Individual phenotypes, however, are also the products of nonadditive genetic effects due to allele interactions within loci and between loci (i.e., dominance and epistasis, respectively), combined with sources of environmental variation (e.g., maternal influences on offspring beyond gene transmission) introduced in early development (Lynch & Walsh, 1998).

Unlike additive genetic effects, nonadditive genetic effects on phenotype depend on genetic backgrounds that are continuously reshuffled by sex and recombination (Wolak & Keller, 2014). Their lack of heritability in the usual sense has made their evolutionary role uncertain, despite considerable theoretical attention (e.g., Barton & Turelli, 2004; Keightley, 1996; Wade & Goodnight, 1998), and underpinned Fisher's (1958) argument that they are irrelevant if populations are assumed to be infinitely large and randomly mating (Wade & Goodnight, 1998). Natural populations, however, often violate these assumptions. In such cases, nonadditive genetic effects can have important effects on evolutionary processes, for example, by creating peaks and valleys on the adaptive landscapes that populations traverse (Peck, Ellner, & Gould, 1998; Wade & Goodnight, 1998; Wright, 1931), by contributing to inbreeding depression (Fenster, Galloway, & Chao, 1997), or by converting to additive genetic variance during population bottlenecks (Cheverud et al., 1999; Goodnight, 1988; Wang, Caballero, Keightley, & Hill, 1998). Nonadditive genetic effects may especially influence adaptive divergence in response to environmental change (Carroll, 2007; Hendry, 2013; Roff & Emerson, 2006). For instance, a diverse range of dominance and epistatic effects were the basis of rapid divergence between soapberry‐bug populations following a change in host plant (Carroll, Dingle, Famula, & Fox, 2001; Carroll, Dingle, & Famula, 2003; see other examples in Bernatchez et al. 2010, and Berner et al. 2011). Indeed, nonadditive genetic effects have been shown to contribute significantly to population differentiation in various aspects of life history and morphology (Roff & Emerson, 2006). In contrast, their contribution to traits of evolutionary interest, and fitness especially, within natural populations remains poorly understood (Sztepanacz & Blows, 2015).

Similarly, the role of maternal environmental effects in evolutionary processes has often been overlooked due to the difficulty of estimating these effects reliably, or because they were traditionally viewed as little more than a nuisance source of variance. Nonetheless, they are now recognized as key influences on offspring fitness (Marshall & Uller, 2007; Rasanen & Kruuk, 2007). Several studies have shown that mothers exposed to a particular type of environmental stress go on to produce offspring with enhanced performance under that stress (Agrawal, Laforsch, & Tollrian, 1999; Johnsen, Daehlen, Ostreng, & Skroppa, 2005; Parker et al., 2012). Other studies, however, have found that stressed mothers go on to produce lower‐quality offspring relative to unstressed mothers (Huxman, Hamerlynck, Jordan, Salsman, & Smith, 1998; Moran, Dias, & Marshall, 2010; Shama & Wegner, 2014). These conflicting results may reflect the degrees to which mothers can predict the environments of offspring (Burgess & Marshall, 2014; Uller, Nakagawa, & English, 2013). In the longer term, Kirkpatrick and Lande (1989) showed that the evolution of maternally influenced traits is facilitated when the selective environments of parents and offspring match, but retarded when they do not (Kirkpatrick & Lande, 1989). Consequently, predicting the evolutionary consequences of maternal effects remains an ongoing challenge, requiring a clearer understanding of how maternal environmental effects, partitioned from genetic effects, contribute to offspring fitness.

The limited evidence available suggests that nonadditive genetic and maternal environmental effects can be sensitive to environmental stress (Blows & Sokolowski, 1995; Kelly, Padilla‐Gamiño, & Hofmann, 2013; Rasanen & Kruuk, 2007), which creates the ecological context (e.g., smaller, subdivided populations, stronger selection, and reduced gene flow) that give the former, especially, greater evolutionary relevance (Wade, 2002; Wade & Goodnight, 1998). For instance, thermal stress altered the expression of nonadditive genetic variance for morphological traits in the field cricket, *Teleogryllus oceanicus* (Nystrand, Dowling, & Simmons, 2011) and for larval hatching success in the sea urchin, *Heliocidaris erythrogramma* (Lymbery & Evans, 2013). Conversely, Foo, Dworjanyn, Poore, and Byrne (2012) found no effects of temperature or pH on nonadditive genetic effects on embryonic development in the sea urchin, *Centrostephanus rodgersii*, nor were maternal effects on development sensitive to CO_2_ in another sea urchin, *Strongylocentrotus franciscanus*, or the mussel, *Mytilus trossulus* (Sunday, Crim, Harley, & Hart, 2011). Furthermore, nonadditive genetic and maternal effects on fitness in one environment can have fitness effects in other environments, which may constrain or accelerate adaptive divergence across them (e.g., Wade, 2000). For example, maternal effects in the marine bryozoan, *Bugula neritina,* increased offspring fitness in high‐pollution and high‐predation environments but decreased fitness under salinity stress (Moran et al., 2010). To our knowledge, however, no study has formally evaluated cross‐environment covariation in nonadditive genetic and maternal environmental effects. Hence, we still know very little about their contributions to fitness, the stability of these contributions under environmental change, and whether their covariation across environments could mitigate or exacerbate the fitness consequences of environmental change.

Here, applying a quantitative genetic breeding design to the ecologically important marine tubeworm, *Galeolaria caespitosa* (henceforth referred to by genus name), we evaluated the contributions of nonadditive genetic effects, and maternal environmental effects, to phenotypic variation in fitness (measured as larval survival) across multiple thermal environments. Typical of free‐spawning marine invertebrates, which shed sperm and eggs into the sea to fuse externally, *Galeolaria*'s life cycle includes a free swimming larval stage and a sessile adult stage (Jackson & Strathmann, 1981; Marshall & Evans, 2005). We focused on larval survival as our fitness measure because free‐spawned larvae are more vulnerable than adults to environmental stress, especially relative to species that brood their young (Byrne, 2011; Jackson & Strathmann, 1981; Marshall & Morgan, 2011). For many marine organisms, therefore, survival at this early stage of the life cycle will be a critical bottleneck in the persistence of future populations. *Galeolaria*'s free‐spawning nature also makes it ideal for exploring larval vulnerability to environmental stress using cross‐classified breeding designs, whereby the subdivision of ejaculates and egg clutches allows males to be mated with multiple females and vice versa (Galletly, Blows, & Marshall, 2007; Munday et al., 2013). Using such a design, we decomposed phenotypic variance in larval survival within and across thermal environments into its nonadditive genetic and maternal environmental components (partitioned from additive genetic effects; see Chirgwin, Monro, Sgro, & Marshall, 2015). Our goal was to understand the relative magnitudes of these often‐neglected sources of fitness variation and their potential consequences for population and evolutionary dynamics under global change.

## 2 Materials and methods

### 2.1 Study species and collection site

*Galeolaria* is an intertidal tubeworm common to South Eastern Australia. The adult stage plays an important ecological role in intertidal areas, forming high‐density colonies that provide habitat for unique endemic communities (Bulleri, Chapman, & Underwood, 2005; Edgar, 2000). We sampled adult *Galeolaria* from an intertidal population at Brighton Marina, Victoria (37°540S 144°590E). The population spawns year‐round and experiences water temperatures ranging from 8 to 25°C, with a mean of ~17°C and a typical maximum of ~22°C. In the previous 15 years, temperatures exceeded 24.5°C for only 6 days (3 days each in January 2013 and 2014). As an intertidal species, however, adults and larvae may experience more extreme temperatures in rockpools at low tide. We sampled the population across two periods (May–August 2013 and February–April 2014). Adults were transported in insulated aquaria to a controlled temperature room at Monash University, Clayton, where they were housed in separate aquaria according to collection date. To reduce the effect of variation in parental environment among collection dates, all adults were acclimatized for 2–3 weeks at ~16°C before their gametes were collected.

### 2.2 Gamete collection and fertilization protocol

Each mature adult was extracted from its calcareous tube and placed into a petri dish of fresh seawater. Individuals began spawning eggs or sperm within 10 s of extraction, at which point gametes were collected. All seawater used during gamete collection and subsequent fertilizations was 17°C, filtered to 0.22 μm and pasteurized.

Following gamete collection, we diluted sperm with seawater to a concentration of 4 × 10^6^ cells per ml (pilot studies showed that fertilization success was maximized at this concentration, before declining at higher concentrations due to polyspermy). As the less abundant gamete, egg concentration has little influence on fertilization success (Levin, Zhu, & Creed, 1991), so we simply extracted all available eggs per female and diluted them to 1.2 ml in seawater. We subsequently added 0.1 ml of the dilute sperm solution to 0.1 ml of the egg solution, doing so three times at 10 min intervals. This gradual addition of sperm was performed to reduce the likelihood of multiple sperm fertilizing the same egg (polyspermy), and to maximize the total fertilization success of each male–female cross (Styan, 1998). The resulting gamete solution was left for 1 hr and then rinsed twice through 0.25 μm Nitex mesh to remove excess sperm.

### 2.3 Cross‐classified (North Carolina II) breeding design and survival assays

**Figure 1:**
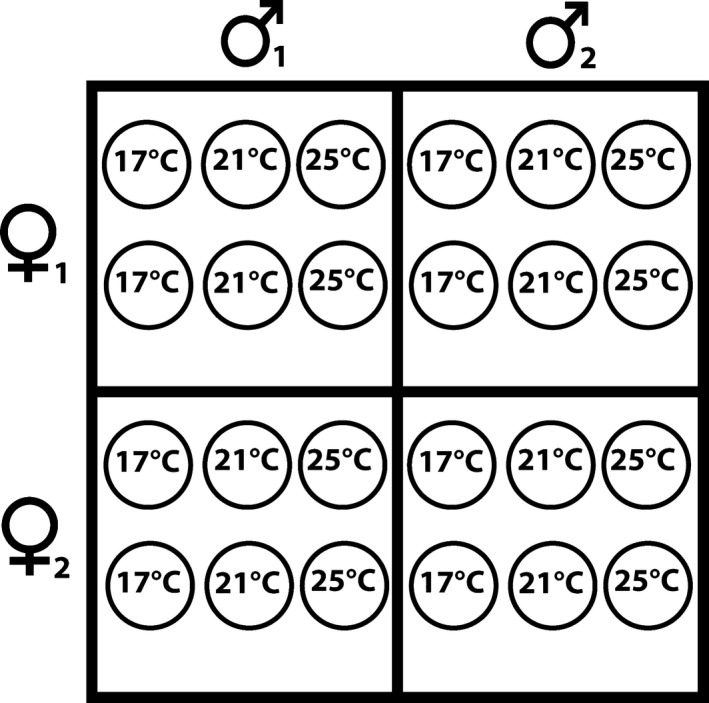
A single block of the North Carolina II breeding design used to estimate parental effects across thermal environments. For each block, eggs from two individual dams were crossed with sperm from two individual sires. Each cross was replicated by six separate fertilizations. Fertilized eggs were then assigned to one of the three temperature treatments (17, 21 or 25°C) so that each sire–dam combination was replicated twice per treatment.

Using the fertilization protocol above, sperm and eggs of *Galeolaria* were crossed according to the North Carolina II (NCII) breeding design (Lynch & Walsh, 1998). Our design consisted of 51 replicate NCII blocks. Each block was the product of sperm from two sires crossed with eggs from two dams, yielding four parental combinations per block (Figure 1). Each parental combination was replicated six times per block, with each of the 24 replicates comprising an independent fertilization (Figure 1).

Approximately 2 hr postfertilization, ~25 normally cleaving embryos were collected from each of the 24 replicates per block and placed in a 1.5‐ml test tube with filtered and sterilized seawater. The percentage of normally cleaving embryos was ~70%–80% per replicate. Each test tube of embryos was randomly assigned to one of the three thermal environments (17, 21 or 25°C), such that each parental combination was replicated twice per temperature (Figure 1). Note that thermal environment was not manipulated during fertilization because gamete environment is known to influence larval performance in other externally fertilizing species, including another *Galeolaria* species (Crean, Dwyer, & Marshall, 2012; Ritchie & Marshall, 2013; White, Mullineaux, McCorkle, & Cohen, 2014). Instead, we manipulated temperature postfertilization to isolate sire and dam effects (which are used to estimate the importance of nongenetic and maternal environmental effects) on larval survival from environmental effects on gametes. After a 48‐hr incubation period, we scored whether larvae had successfully survived to the trochophore stage, which previous ecotoxicological studies have identified as the most sensitive and reliable indicator of larval tolerance to stress (Ross & Bidwell, 2001). Hence, larval survival, quantified as the number of normally developing trochophores, was the ecologically relevant measure of fitness used in our study. Overall, we scored over 30,000 embryos from 204 families.

### 2.4 Manipulation of thermal environment

Thermal environments were chosen to represent (i) the mean annual sea‐surface temperature at our collection site (17°C); (ii) a low‐to‐moderate rise from the mean annual sea‐surface temperature (21°C); and (iii) the highest sea‐surface temperature recorded in the past 12 months at our collection site (25°C; CSIRO, 2014). Ecologically, our elevated temperatures represent the typical summer conditions (21°C), plus a temperature that is currently rare but likely to become more common in future years (25°C). As such, they are likely to be a good reflection of near‐future thermal stress for our study population. All thermal environments were implemented by incubating test tubes of embryos in mini heating dry‐baths. For the two elevated temperatures, test tubes of embryos were gradually heated to the desired temperature over ~20 min. Each thermal environment was maintained within 0.2°C of its nominal temperature throughout the 48‐hr incubation period.

### 2.5 Statistical analyses

We used a multivariate linear mixed model to investigate how temperature influenced the expression of nonadditive genetic and maternal environmental effects (partitioned from additive genetic variance; see Chirgwin et al., 2015) on larval survival. The model was fitted with restricted maximum likelihood in the MIXED procedure of SAS 9.3 (SAS Institute, Cary, NC). Specifically, the model was:

$$\mathrm{larval}\;\mathrm{survival}=\mathrm{XB}+Z_{s}\sigma_{s}^{2}+Z_{d}\sigma_{d}^{2}+Z_{sd}\sigma_{sd}^{2}+\varepsilon$$

where **X** was the design matrix for the fixed effects (**B**) of temperature and block, and **Z**_*d*_ *,* **Z**_*s*_, and **Z**_*sd*_ were design matrices for the random effects of sire (σ^2^_*s*_), dam (σ^2^_*d*_), and sire × dam interaction (σ^2^_*sd*_), respectively. Each random effect (and the residual term, **ε**) was an unstructured matrix containing the variances within, and covariances across, the three thermal environments. The model also included sampling period (May–August 2013 vs February–April 2014) as another fixed factor, plus a separate residual matrix for each period. Note, however, that sampling period did not alter the expression of parental effects (i.e., the contributions of sires, dams, and sire x dam interactions to larval survival) across temperatures, so is not considered further here.

We then converted the observational (co)variance components (σ^2^_*s*_, σ^2^_*d*_ and σ^2^_*sd*_) obtained from this model into causal components of additive genetic variance (σ^2^_*A*_), nonadditive genetic variance (σ^2^_*I*_), and maternal environmental variance (σ^2^_*M*_), using the following standard equations (Fry, 2004):

$$\sigma_{A}^{2}=4\sigma_{s}^{2}$$

$$\sigma_{I}^{2}=4\sigma_{sd}^{2}$$

$$\sigma_{M}^{2}=\sigma_{d}^{2}-\sigma_{s}^{2}.$$

Note that σ^2^_*I*_ represents the combined effects of dominance and epistasis, which the experimental design did not allow us to disentangle. Note also that the estimate of σ^2^_*M*_ assumes that dams and sires have the same additive genetic contribution to their offspring (Fry, 2004).

We used an *F*‐test to examine temperature effects on larval survival and used standard log‐likelihood ratio tests to examine the significance of all random effects. We tested the overall contribution of nonadditive genetic effects to larval survival by comparing the full model to a reduced model that constrained all sire x dam (co)variances to be zero. We tested the overall contribution of maternal environmental effects by comparing the full model to a reduced model that constrained dam (co)variances and sire (co)variances to be equal (Fry, 2004). For each set of effects, we also tested whether individual (co)variance components differed to zero. To visualize the total contribution of parental effects to variance in larval survival at each temperature, we plotted the sum of each additive genetic, nonadditive genetic, and maternal environmental variance against the residual variance. Next, to visualize the relative contributions of parental effects at each temperature, we plotted the proportional effects of additive genetic, nonadditive genetic, and maternal environmental variance against each other.

Readers should note that the data set analyzed here was also the source for Chirgwin et al. (2015), which nonetheless has limited overlap with this study. That study focused on the distribution of additive genetic variance in multivariate space, reporting only the percentages of variance in larval survival contributed by dams (combining genetic and environmental effects) and sire x dam interactions within single environments. Here, we focus explicitly on maternal environmental effects (partitioned from genetic effects) and present new multivariate analyses that offer novel insights into cross‐environment covariation in both those and nonadditive genetic effects. Sire effects from Chirgwin et al. (2015) are re‐included here (converted from σ^2^_*s*_ to σ^2^_*A*_) as a benchmark for evaluating the magnitudes of other parental influences on fitness.

## 3 Results

### 3.1 Larval survival across thermal environments

**Figure 2:**
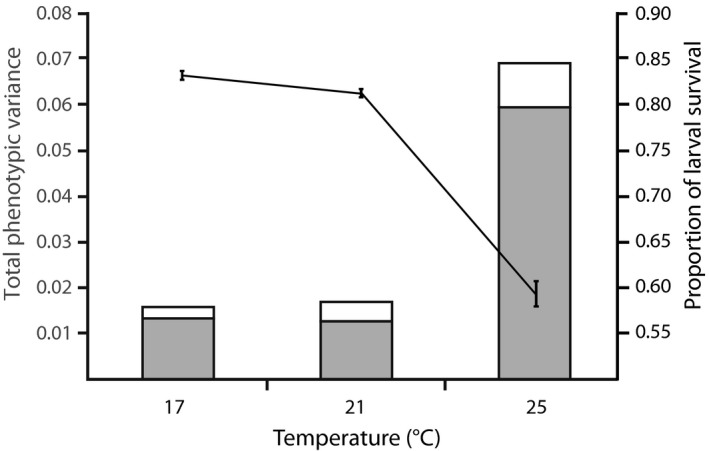
The left axis and columns show the amount of phenotypic variance in larval survival explained by parental effects (summed across additive genetic, nonadditive genetic and maternal environmental effects, in black) relative to unexplained variance (white) in at each temperature. The right axis and gray line show the mean survival (±SE) of larvae at each temperature.

Larval survival declined across thermal environments (*F*_2,153_ = 104.76, *p *<* *.001; Figure 2). Note that this test differs to Chirgwin et al. (2015) because it is conditioned on a different specification of random effects (biological inferences are unchanged). Post hoc tests confirmed that survival differed significantly between each pair of temperatures, with survival at the highest temperature approximately two‐thirds of that at the lowest temperature.

### 3.2 Nonadditive genetic and maternal environmental effects within thermal environments

Overall, the total variance in larval survival was similar at the two coolest temperatures (17 and 21°C), but was amplified fourfold by the warmest temperature (25°C; Figure 2). As the unexplained (residual) variance in survival remained more‐or‐less similar among environments, accounting for no more than 20% of the total variance in any single one, this fourfold increase represents a temperature‐induced change in the expression of parental effects (summed across additive genetic, nonadditive genetic, and maternal environmental effects).

**Figure 3:**
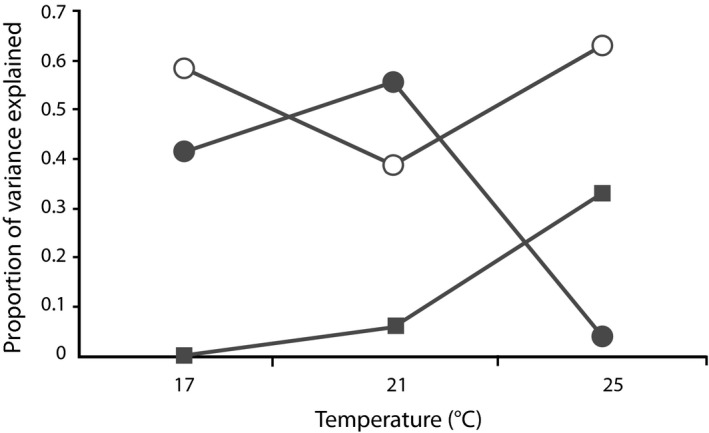
The relative proportions of the total phenotypic variance in larval survival explained by each source of parental effect: additive genetic effects (gray circles), nonadditive genetic effects (white circles), and maternal environmental effects (gray squares).

A closer look at the relative contributions of each parental influence (Figure 3) revealed that nonadditive genetic and maternal environmental effects drove the greater variability of larval survival at 25°C. Indeed, they explained 96% of all parental effects on survival at this temperature (rising from 58% at 17°C and 44% at 21°C), whereas additive genetic effects were similar in magnitude, or relatively greater, at the less extreme temperatures. Specifically, nonadditive genetic effects on survival were significant in all environments (Table 1), accounting for roughly two‐thirds of the parental effects expressed at 17 and 25°C, and nearly half of them expressed at 21°C (Figure 3). In contrast, maternal environmental effects on survival were significant at 25°C only (Table 1), accounting for roughly a third of the parental effects expressed in that environment (Figure 3), but contributing relatively little to their expression at 17 and 21°C (Figure 3).

**Table 1:** The variances and covariances of parental effects on larval survival within and across thermal environments: a) nonadditive genetic effects, b) maternal effects (reported as 0 when the dam variance was less than the corresponding sire variance; see text for details), and c) additive genetic effects. Within‐environment variances are in bold on the diagonal and cross‐environment covariances are in italics below the diagonal (*p <* *.05)

	17°C	21°C	25°C
a) Nonadditive genetic effects			
17°C	0.007788*		
21°C	0.004776*	0.004912*	
25°C	*−0.00096*	0.010694*	0.037544*
b) Maternal effects			
17°C	**0**		
21°C	*0*	**0.000727**	
25°C	*0*	*0.001029*	0.019622*
c) Additive genetic effects^a^			
17°C	0.005536*		
21°C	0.005016*	0.007056*	
25°C	0.008088*	*0.002244*	**0.002352**

a Reproduced from table 2 in Chirgwin et al., 2015, converted to causal components (see text for details).

### 3.3 Cross‐environment covariation in nonadditive genetic and maternal environmental effects

Nonadditive genetic effects on larval survival covaried significantly across adjacent thermal environments (i.e., between 17 and 21°C, and between 21 and 25°C, but not between 17 to 25°C; Table 1a). Such covariation was positive in both cases (Table 1), indicating that parental combinations that performed relatively well (or poorly) at one temperature tended to also do so at the next warmest temperature. In contrast, maternal environmental effects on survival were decoupled across environments (Table 1b). Hence, whether or not a given maternal environment was beneficial to offspring at one temperature had no bearing on offspring survival at other temperatures (although this lack of covariation might also reflect the overall weakness of maternal environmental effects in our study).

## 4 Discussion

Additive genetic variance is critical for evolutionary responses to global change, yet is not the only source of fitness variation available for selection in natural populations. While the evolutionary roles of nonadditive genetic and maternal environmental effects remain controversial, theory and data argue that they can substantially alter evolutionary trajectories, as well as magnitudes and effects of gene flow (Dey, Proulx, & Teotónio, 2016; Hendry, 2013; Kirkpatrick & Lande, 1989; Rasanen & Kruuk, 2007; Wade, 2002; Wang et al., 1998). Little is known, however, of their relative contributions to fitness variation in natural populations, and even less of their multivariate, multi‐environment impacts that might exacerbate or ameliorate global‐change stressors. We found that nonadditive genetic and maternal environmental effects on larval survival in *Galeolaria* are nontrivial and environment dependent, explaining no less than 44% of parental effects on survival in any environment, and 96% of parental effects in the most stressful one. In Chirgwin et al. (2015), we examined the fraction of variance in larval survival explained by additive genetic effects; here, we consider the possible fitness consequences of the other 96%.

Our results imply that nonadditive genetic and maternal environmental effects may increasingly influence the population and evolutionary dynamics of marine free‐spawners, such as *Galeolaria*, as water temperatures rise with global change. Nonadditive genetic effects accounted for large proportions (39%–63%) of parental effects on larval survival across thermal environments ranging from present‐day conditions to those predicted in the future, while maternal environmental effects accounted for considerable variance (33%) in the warmest one. Previously, we showed that *Galeolaria* harbors significant levels of additive genetic variance in larval survival across these environments that may facilitate adaptation to future warming (Chirgwin et al., 2015). However, adaptation to environmental change requires more than additive genetic variance alone: That populations must also persist while they accumulate alleles that are beneficial in the changed conditions (Bell, 2013; Gomulkiewicz & Holt, 1995) warrants attention to other sources of fitness variation that may aid persistence and contribute to evolutionary processes (Merilä & Sheldon, 1999). The adaptive value of nonadditive genetic and maternal environmental effects is often discounted on grounds (i.e., that they are small and transient in nature) that are increasingly disputed (Hansen, 2013; Uller et al., 2013). Here, their effects on larval survival in *Galeolaria* give them the potential to aid persistence in the face of future warming and thermal variability, and lead to evolutionary dynamics that differ to those predicted by additive genetic variance alone.

Nonadditive genetic effects on fitness were strongly temperature dependent, being similar in magnitude to additive genetic effects at 17 and 21°C, but explaining the majority of fitness variation at 25°C. Previous studies have detected similar patterns, finding that environmental stress reduces additive genetic variance (Bubliy & Loeschcke, 2002; Galletly et al., 2007) and increases nonadditive genetic variance (Blows & Sokolowski, 1995; Jinks, Jean, & Pooni, 1973). However, other studies have found stress to have the opposite effect, or little effect at all (Hoffmann & Parsons, 1991; Pakkasmaa, Merila, & O'Hara, 2003). One reason for this discrepancy could be that different stress levels impose different strengths of selection on focal traits. Crnokrak and Roff (1995), for example, reported that traits under stronger selection harbor higher levels of nonadditive genetic variance relative to weakly selected traits (see also Hoffmann & Parsons, 1991). Currently, however, empirical tests remain too few to allow for broad generalizations about the environment dependence of nonadditive genetic effects. Their evaluation across a greater range of traits and stressors would greatly enhance our understanding of this issue.

That nonadditive genetic effects were amplified at the highest temperature implies that they may become progressively important to population and evolutionary dynamics under future warming. This is essentially because such effects are most influential in small, subdivided populations incurring strong selection (Wade, 2002), which are increasingly associated with global change (Gienapp et al., 2008; Jump & Penuelas, 2005; Moller, Rubolini, & Lehikoinen, 2008). Warming‐driven declines in population size, for example, could see greater conversion of nonadditive variance into additive variance (Barton & Turelli, 2004; Cheverud et al., 1999; Goodnight, 1988; Wang et al., 1998), although van Heerwaarden, Willi, Kristensen, and Hoffmann (2008) showed that increases in the latter during population bottlenecks do not necessarily improve adaptive capacity in *Drosophila*. Alternatively, greater expression of nonadditive genetic effects under warming might not only hinder adaptive capacity by masking favorable or unfavorable alleles from selection, but also hinder the erosion of additive genetic variance in doing so (Crnokrak & Roff, 1995). Regardless, the presence of substantial nonadditive genetic effects on fitness has implications for how managers use genetic translocations to maintain population genetic diversity (Edmands, 2007; Tallmon, Luikart, & Waples, 2004). If nonadditive genetic effects rely on allele interactions that have evolved within specific populations, then translocations between populations may in principle cause outbreeding depression due to hybrid breakdown (Edmands, 1999; Fenster et al., 1997), although in practice there is little evidence of this phenomenon (Frankham, 2015). Further work exploring how nonadditive genetic effects on fitness influence the efficacy of genetic translocations could provide managers with crucial information for protecting populations from future environmental change.

While maternal environmental effects had little impact on the survival of *Galeolaria* larvae at lower temperatures, their greater expression at the highest temperature suggests that they may also influence how marine ectotherms respond to warming waters. There is growing awareness that such effects can contribute to adaptation in natural populations, especially when maternal and offspring environments are positively correlated (Burgess & Marshall, 2014; Dey et al., 2016; Salinas & Munch, 2012; Shama, 2015; Uller et al., 2013). For instance, Donelson, Munday, McCormick, and Pitcher (2012) found that damselfish (*Acanthochromis polyacanthus*) exposed to thermal stress produce offspring with superior thermal tolerance relative to offspring of unexposed parents. Other studies, however, have shown that stressful parental environments can lower offspring quality (Guillaume, Monro, & Marshall, 2016; Huxman et al., 1998; Lane, Campanati, Dupont, & Thiyagarajan, 2015; Shama & Wegner, 2014). In our study, maternal environmental effects on survival were unlikely to have been caused by past environmental conditions, as all mothers came from the same collection site and were acclimatized before use. Although the mechanism remains unclear, our results nonetheless indicate that maternal environmental effects can potentially influence the viability of marine populations in warming waters and should therefore be considered in future management strategies.

As global change is predicted to increase both the mean and variability of water temperatures, it is important to understand the capacity for populations to withstand and adapt to multiple temperatures simultaneously. To explore how nonadditive genetic and maternal environmental effects on larval survival may affect *Galeolaria*'s persistence in variable thermal environments, we estimated covariation in these effects across all environments in which survival was assayed. Encouragingly, we found that nonadditive genetic effects on survival covaried positively across environments, in contrast to recent suggestions that the exposure of unfavorable nonadditive effects by thermal stress (Eads, Mitchell, & Evans, 2012; Lymbery & Evans, 2013) may lead to fitness trade‐offs across stress levels. Here, however, we found no evidence of such trade‐offs. Instead, parental combinations that produce a selective advantage in one thermal environment may also do so in other environments, thereby buffering *Galeolaria* against temperature variation.

The question remains of whether cross‐environment covariation in nonadditive genetic effects can influence thermal adaptation beyond such buffering – for example, if parental combinations that perform well under ambient heat stress are primed to exploit more extreme environments (e.g., higher in the intertidal) or contribute disproportionately to the gene pool after warming‐driven declines in population size. These scenarios, of course, assume that nonadditive genetic effects are to an extent stable across generations. However, growing evidence of their effects on population differentiation following environmental change (Carroll, 2007; Hendry, 2013) suggests some capacity for this to occur, particularly when populations undergo decline or subdivision (Roff & Emerson, 2006; Wade, 2002). If such is the case for *Galeolaria*, then cross‐environment covariation in nonadditive genetic effects on fitness could potentially influence the evolutionary dynamics of our study population under global change. Given how little is currently known about the generality of this phenomenon, we suggest that estimates of such covariation warrant better characterization and be reported whenever possible in future.

Surprisingly, we found no evidence of maternal environmental effects on survival covaried across temperatures. Thus, maternal environmental effects that conferred either a benefit or burden to offspring survival in one environment had no bearing on offspring performance in any other environment. Consequently, the ability of our study population to withstand greater temperature variability appears unlikely to be facilitated or constrained by cross‐environment correlations in maternal environmental effects on fitness. Nevertheless, such correlations may potentially influence population responses to other global‐change stressors, such as water pH and oxygen concentration (Byrne, 2011; Reusch, 2014), and are worthy of further investigation.

Despite ongoing debate over the evolutionary relevance of nonadditive genetic and maternal environmental effects (Hill, Goddard, & Visscher, 2008; Rasanen & Kruuk, 2007; Uller et al., 2013; Wolak & Keller, 2014), the rapid rate of global change, and its impacts on population size and structure, makes understanding their fitness consequences increasingly important. Overall, we argue that nonadditive genetic and maternal environmental effects may play important roles in population and evolutionary responses of marine species to rising water temperatures. While our goal here was to draw attention to the size and environmental sensitivity of these effects, our work now highlights the need to better incorporate them into predictions of population persistence in changing environments. In particular, there is pressing need for studies that examine the stability of nonadditive genetic and maternal environmental effects across multiple generations (e.g., Dey et al., 2016; van Heerwaarden et al., 2008), that incorporate them into projections of population dynamics (e.g., Coulson, Tuljapurkar, & Childs, 2010), and that consider their effects in multiple or fluctuating environments. Such work is currently rare, but will enhance our ability to forecast the adaptive capacity of populations exposed to global change so they can be managed more efficiently.

## Data archiving statement

Data available from the Dryad Digital Repository: http://dx.doi.org/10.5061/dryad.869cf.
